# Patient Attitudes Toward Individualized Recommendations to Stop Low-Value Colorectal Cancer Screening

**DOI:** 10.1001/jamanetworkopen.2018.5461

**Published:** 2018-12-07

**Authors:** Marc S. Piper, Jennifer K. Maratt, Brian J. Zikmund-Fisher, Carmen Lewis, Jane Forman, Sandeep Vijan, Valbona Metko, Sameer D. Saini

**Affiliations:** 1 Division of Gastroenterology, Department of Internal Medicine, Providence-Providence Park Hospital, Michigan State University College of Human Medicine, Southfield; 2 Division of Gastroenterology, Department of Internal Medicine, University of Michigan, Ann Arbor; 3 Department of Health Behavior and Health Education, University of Michigan School of Public Health, Ann Arbor; 4 Division of General Medicine, Department of Internal Medicine, University of Michigan, Ann Arbor; 5 Division of General Internal Medicine, Department of Internal Medicine, University of Colorado, Aurora; 6 Veterans Affairs (VA) Health Services Research and Development Service Center for Clinical Management Research, VA Ann Arbor Healthcare System, Ann Arbor, Michigan

## Abstract

**Importance:**

Guidelines for colorectal cancer (CRC) screening recommend an individualized approach in older adults that is informed by consideration of life expectancy and cancer risk. However, little is known about how patients perceive individualized screening recommendations.

**Objective:**

To assess veterans’ attitudes toward and comfort with cessation of low-value CRC screening (defined as screening in a patient for whom the benefit is expected to be small based on quantitative estimates from hypothetical risk calculators).

**Design, Setting, and Participants:**

This survey study included patients older than 50 years who had undergone prior screening colonoscopy with normal results at the Veterans Affairs Ann Arbor Healthcare System. A total of 1500 surveys were mailed to potential participants from November 1, 2010, to January 1, 2012. Survey data were analyzed from January 1, 2016, to December 31, 2017.

**Main Outcomes and Measures:**

Response to the question, “If you personally had serious health problems that were likely to shorten your life and your doctor did not think screening would be of much benefit based on the calculator, how comfortable would you be with not getting any more screening colonoscopies?”

**Results:**

Of the 1500 surveys mailed, 85 were returned to sender, leaving 1415 potential respondents; 1054 of these respondents (median age range, 60-69 years; 884 [85.9%] white and 965 [94.2%] male) completed the survey (response rate, 74.5%). A total of 300 (28.7%) were not at all comfortable with cessation of low-value CRC screening, and 509 (49.3%) thought that age should never be used to decide when to stop screening. In addition, 332 (31.7%) thought it was not at all reasonable to use life expectancy calculators, and 255 (24.3%) thought it was not at all reasonable to use CRC risk calculators to guide these decisions. In ordered logistic regression analysis, factors associated with more comfort with screening cessation were (1) higher trust in physician (odds ratio [OR], 1.19; 95% CI, 1.07-1.32), (2) higher perceived health status (OR, 1.41; 95% CI, 1.23-1.61), and (3) higher barriers to screening (OR, 1.20; 95% CI, 1.11-1.30). Factors that were associated with less comfort with screening cessation included (1) greater perceived effectiveness of screening (OR, 0.86; 95% CI, 0.80-0.94) and (2) greater perceived threat of CRC (OR, 0.81; 95% CI, 0.73-0.89).

**Conclusions and Relevance:**

The findings suggest that many veterans have strong preferences against screening cessation even when given detailed information about why the benefit may be low. Efforts to tailor screening recommendations may be met by resistance unless they are accompanied by efforts to address underlying perceptions about the benefit of screening.

## Key Points

**Question:**

How comfortable are patients with stopping colorectal cancer screening when the benefit is expected to be low for them personally, and what factors are associated with comfort in stopping?

**Findings:**

In this survey study of patients in the Veterans Affairs health care system, among 1054 respondents, 29% were not at all comfortable with stopping colorectal cancer screening when the benefit was expected to be low. Factors associated with more comfort in stopping screening were higher trust in physician, higher perceived health status, and higher perceived barriers to screening, and factors associated with less comfort were greater perceived effectiveness of screening and greater perceived threat of colorectal cancer.

**Meaning:**

The findings suggest that patients would be uncomfortable stopping low-value colorectal cancer screening despite a physician’s individualized recommendation to stop because of low benefit.

## Teaser

This survey study assesses patient attitudes and preferences about cessation of low-value colorectal cancer screening and their overall comfort using age, life expectancy calculators, and colorectal cancer risk calculators in helping make individualized screening decisions.

## Introduction

Colorectal cancer (CRC) screening is a widely recommended and highly valued preventive service that has traditionally been underused.^1,2,3^ During the past several decades, organized efforts to address screening underuse have shown success, with steady increases in use of screening.^4^ However, one unintended consequence has been overuse of low-value CRC screening, particularly in older and less healthy patients who have limited life expectancy.^1,5,6,7,8,9^

Low-value screening can be defined in a variety of ways. Fundamentally, low-value screening is any screening test that is unlikely to provide benefit to a patient in terms of quality or quantity of life. In the context of older adults with previous colonoscopies with normal results who are at low risk for CRC, the harms of screening (including unnecessary discomfort, diminished quality of life, and procedure-related harm) are likely to outweigh the benefits.^10,11,12,13^

Because screening benefit varies from patient to patient, experts recommend that screening decisions be individualized in older adults. Screening benefit can be estimated using individual patient characteristics, such as age, life expectancy, and cancer risk.^14,15,16^ For example, the US Preventive Services Task Force revised recommendations for CRC screening suggest that screening be individualized for those aged 76 to 85 years (grade C) according to overall health (a measure of life expectancy), screening history (a measure of CRC risk), and patient preferences.^17^

However, little is known about patient attitudes toward individualized screening. Therefore, we examined patients’ attitudes and preferences toward cessation of low-value CRC screening. In addition, we assessed their overall comfort with use of age, life expectancy calculators, and CRC risk calculators in helping make individualized screening decisions.

## Methods

We performed a survey study of veterans with normal results on average-risk screening colonoscopy performed at the Veterans Affairs Ann Arbor Healthcare System (VAAAHS). The VAAAHS is a tertiary referral facility that provides care to more than 65 000 veterans residing in Michigan and northwestern Ohio. The health care system performs approximately 5000 gastrointestinal endoscopies per year for veterans from across this catchment area. The institutional review board of the VAAAHS approved this study. Participant informed consent was implied with return of the survey. All data were deidentified. This study followed the American Association for Public Opinion Research (AAPOR) reporting guideline.^18^

### Survey Design

Survey development was informed by semistructured interviews on attitudes toward cessation of CRC screening, conducted with patients from November 1, 2010, to January 1, 2012. Survey data were analyzed from January 1, 2016, to December 31, 2017. The survey questions were created with the assistance of a survey expert (B.J.Z.-F.) and pretested on a pilot sample of 10 patients using a think-aloud approach to assess and improve the understandability of survey items before wider distribution. The final survey comprised 42 questions that took approximately 15 to 20 minutes to complete. A total of 1500 surveys were mailed.

In the survey, we presented each participant with a detailed, hypothetical scenario of a patient-physician clinical encounter. The scenario began with an explanation of how and why screening is initiated at 50 years of age in average-risk individuals, followed by an explanation of current screening cessation recommendations (stop after 75 years of age in adequately screened individuals). The scenario then turned to the use of risk calculators for life expectancy, CRC risk, and overall screening benefit. With each concept, the respondent was asked to rate how reasonable they thought it was to use such information to make screening decisions (on a 7-point unidirectional Likert-type scale, with 1 indicating “not at all reasonable” and 7 indicating “extremely reasonable”). To assess attitudes toward low-value CRC screening cessation (primary outcome), respondents were asked, “If you personally had serious health problems that were likely to shorten your life and your doctor did not think screening would be of much benefit based on the calculator, how comfortable would you be with not getting any more screening colonoscopies?” All responses were recorded on a unidirectional 7-point Likert-type scale, with 1 indicating “not at all comfortable” and 7 indicating “extremely comfortable.” We also assessed secondary outcomes, such as veterans’ comfort with the use of age as the basis of when to start and stop CRC screening, along with their comfort with the use of life expectancy and risk calculators in guiding CRC screening decisions.

We collected baseline information on demographics, which included age, sex, race/ethnicity, marital status, highest level of education, self-reported health status, and health literacy (using a validated scale by Chew et al^19^). In addition, we assessed veterans’ trust in their physician, prior experience with CRC screening, and health belief model constructs, which included measures of perceived susceptibility to CRC, threat of CRC, effectiveness of colonoscopy, and barriers to completing colonoscopy.^20^ The final survey instrument is available in the eAppendix in the Supplement.

### Population and Sampling

We identified participants through the electronic endoscopic database at the VAAAHS. We included veterans who met the following criteria: (1) age of 50 years or older and (2) normal results of prior complete, average-risk screening colonoscopy with adequate bowel preparation performed between January 1, 2007, and December 31, 2011. We excluded individuals if they had a personal history of adenomas, family history of CRC, or a personal history of inflammatory bowel disease. We mailed the survey in April 2012, with a second mailing to nonresponders 3 weeks later. All potential respondents received a $10 gift card. We used the AAPOR reporting guideline for survey studies to calculate response rate. We considered surveys marked “return to sender” as not eligible for response.^18^

### Statistical Analysis

We report descriptive statistics (means or proportions) and multivariable ordered logistic regression analysis used to identify factors associated with comfort with stopping low-value CRC screening. The primary outcome (dependent variable) in our regression analysis was the response to the question mentioned above. Because the dependent variable was ordered but not continuous, we chose to use multivariable ordered (ordinal) regression rather than linear regression. Independent variables for this regression analysis were selected a priori (ie, we did not conduct a stepwise regression for variable selection). Odds ratios (ORs) were used to represent the association between a 1-point increase in a given independent variable (assuming that all other independent variables are held constant) and comfort with CRC screening cessation. If the 95% CI for an OR did not include 1.0, the OR was considered to be statistically significant at the 5% level (2-tailed *P* < .05). A total of 68 of 1054 cases (6.5%) had missing data and were dropped from the analysis. All analyses were performed using Stata, version 14.1 (StataCorp).

## Results

Of the 1500 surveys mailed, 85 were returned to sender. Of the remaining 1415 potential respondents, 1054 completed the survey (response rate, 74.5%). Respondents were predominately white (884 [85.9%]) and male (965 [94.2%]). The median age range was 60 to 69 years. Most patients (761 [74.0%]) reported their general health as good, very good, or excellent. A total of 294 respondents (28.2%) reported their health literacy as limited or marginal. A total of 512 respondents (49.7%) had completed education beyond high school, and 54 patients (5.2%) had either not graduated high school or earned a vocational or technical degree. Respondent characteristics are detailed in Table 1.

**Table 1.** Baseline Characteristics of 1054 Survey Respondents

Characteristic	Respondents, No. (%)
Age, y	
40-49	1 (0.1)
50-59	272 (26.3)
60-69	587 (56.8)
70-79	140 (13.6)
80-89	33 (3.2)
Sex	
Male	965 (94.2)
Female	59 (5.8)
Race/ethnicity	
White	884 (85.9)
Black	98 (9.5)
Latino	28 (2.7)
Other	19 (1.9)
Health literacy^19^^,^^a^	
Not at all	30 (2.9)
A little bit	60 (5.8)
Somewhat	204 (19.6)
Quite a bit	354 (34.0)
Extremely	394 (37.8)
Self-reported health status	
Poor	42 (4.1)
Fair	225 (21.9)
Good	419 (40.8)
Very Good	281 (27.3)
Excellent	61 (5.9)
Trust in physician^b^	
1	4 (0.4)
2	15 (1.4)
3	29 (2.8)
4	97 (9.3)
5	189 (18.2)
6	397 (38.2)
7	308 (29.6)
Age to stop screening^c^	
<75 y	65 (6.3)
75 y	278 (26.9)
>75 y	180 (17.4)
Should never use age	509 (49.3)

^a^ Patient responses to, “How confident are you filling out medical forms?”

^b^ Patient responses to, “In general, how likely are you to trust your doctor’s medical recommendations?” Question was answered on a Likert-type scale with 1 indicating not at all and 7 indicating extremely.

^c^ Patient responses to, “At what age do you think doctors should stop screening patients who had a normal screening colonoscopy in the past?”

### Attitudes Toward Use of Age in the Decision to Start and Stop CRC Screening

**Figure 1.**
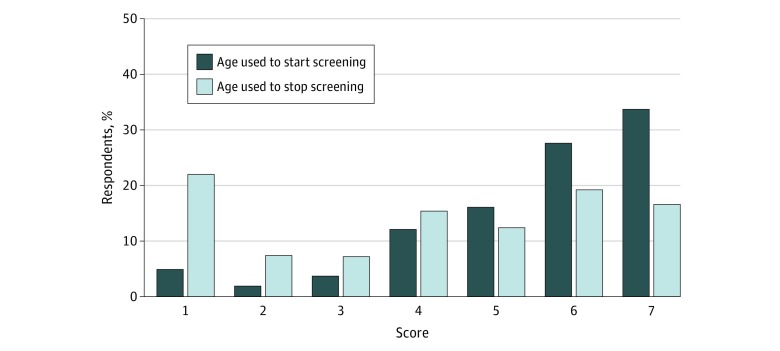
Attitudes Toward Use of Age to Decide When to Start and Stop Colorectal Cancer Screening Proportion of patients’ responses about whether age should be used by physicians to decide when to start and stop screening. Scores were measured on a Likert-type scale with 1 indicating not at all and 7 indicating extremely.

Respondents generally thought that it was reasonable to use age to decide when to start CRC screening. Specifically, 347 of 1031 (33.7%) stated that it was extremely reasonable to use age to decide when to start screening, and 798 (77.4%) responded with 5 or higher on the Likert-type scale that ranged from not at all reasonable to extremely reasonable (Figure 1) (eTable 1 in the Supplement).

However, despite its common use in clinical practice,^21^ respondents were more hesitant about the use of age to decide when to stop screening, with 227 participants (22.0%) responding that it was not at all reasonable and 377 (36.6%) responding with a 3 or less on the Likert-type scale. Overall, veterans thought it was more reasonable to use a uniform starting age than it was to use a uniform stopping age (Figure 1) (eTable 1 in the Supplement). When specifically asked, “At what age do you think doctors should stop screening patients who have had normal results of screening colonoscopy in the past?” 509 of 1032 (49.3%) said physicians should never use age to decide when to stop screening (Table 1).

### Attitudes Toward Life Expectancy and CRC Risk Calculators

**Figure 2.**
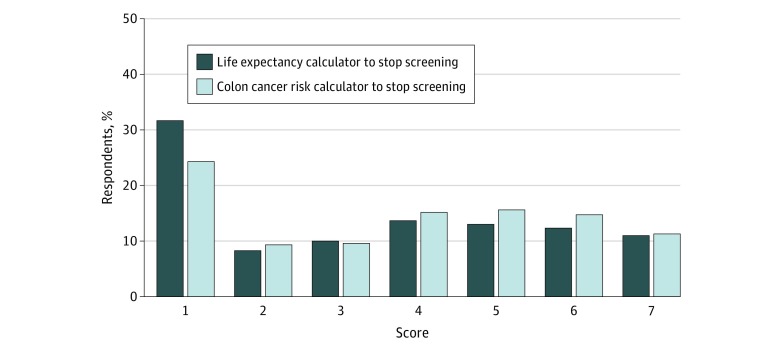
Attitudes Toward Use of Risk Calculators to Inform Colorectal Cancer Screening Decisions Proportion of patients’ responses about whether a life-expectancy calculator or colon cancer risk calculator should be used by physicians to decide when to stop screening. Scores were measured on a Likert-type scale with 1 indicating not at all and 7 indicating extremely.

When respondents were informed of a life expectancy calculator that could be used to guide the decision of whether to stop CRC screening, 332 of 1049 (31.7%) thought it was not at all reasonable to use such a calculator. Similarly, in another scenario that used a CRC risk calculator, 255 of 1049 participants (24.3%) thought it was not at all reasonable to use such a tool (Figure 2) (eTable 2 in the Supplement). In both scenarios, negative attitudes toward calculators were driven by concerns about accuracy (307 of 1049 [29.3%] and 211 of 1048 [20.1%] thought that a life expectancy calculator and a CRC risk calculator would not be at all accurate). In contrast, more than one-third expressed at least some degree of comfort with each calculator (score ≥5 on the 7-point Likert-type scale).

### Attitudes Toward Stopping Low-Value CRC Screening

**Figure 3.**
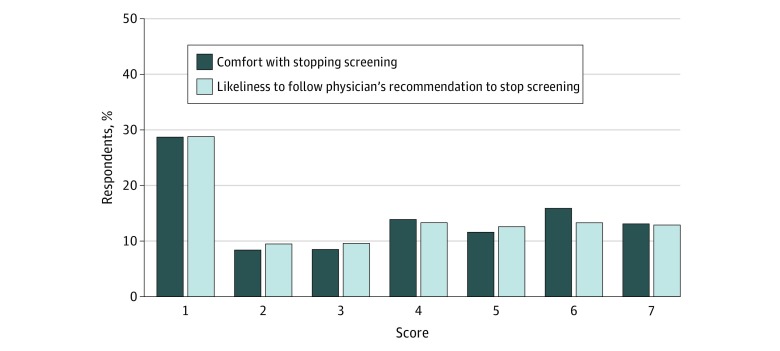
Attitudes Toward Stopping Low-Value Colorectal Cancer Screening and Likelihood of Following Recommendations to Stop Colorectal Cancer Screening Proportion of patients’ responses about comfort with a physician’s decision to stop screening because of the lack of benefit based on the life expectancy calculator and whether the patient would follow the physician’s recommendation to stop screening. Scores were measured on a Likert-type scale with 1 indicating not at all and 7 indicating extremely.

Many respondents were hesitant to stop low-value CRC screening despite poor health and a physician’s recommendation to stop. Specifically, when asked, “If you personally had serious health problems that were likely to shorten your life and your doctor did not think screening would be of much benefit based on the calculator, how comfortable would you be with not getting any more screening colonoscopies?” 300 of 1047 (28.7%) stated that they would be not at all comfortable compared with 137 (13.1%) stating that they would be extremely comfortable (Figure 3 and eTable 3 in the Supplement). In addition, when asked, “How likely do you think you’d be to follow the doctor’s recommendation to stop screening for colon cancer (whether or not you are comfortable with it)?” 298 of 1036 (28.8%) stated that they would not be at all likely to follow this recommendation. In contrast, 424 (40.6%) expressed at least some degree of comfort with screening cessation (score of ≥5 on the 7-point Likert-type scale). Thus, respondents had diverse opinions about screening cessation, although a substantial few had strong opinions about not stopping in the context of low benefit.

### Factors Associated With Comfort With Low-Value Screening Cessation

Because many patients were uncomfortable with screening cessation and were unlikely to follow a physician recommendation to stop screening, we examined factors associated with comfort with screening cessation. In multivariable ordered logistic regression that included demographic factors, health literacy, trust in health care provider, and health belief model constructs, factors that were associated with more comfort with screening cessation included (1) higher trust in physician (OR, 1.19; 95% CI, 1.07-1.32), (2) higher perceived health status (OR, 1.41; 95% CI, 1.23-1.61), and (3) higher barriers to screening (OR, 1.20; 95% CI, 1.11-1.30). Factors that were associated with less comfort with screening cessation included (1) greater perceived effectiveness of screening (OR, 0.86; 95% CI, 0.80-0.94) and (2) greater perceived threat of CRC (OR, 0.81; 95% CI, 0.73-0.89) (Table 2).

**Table 2.** Factors Associated With Comfort With Stopping Low-Value Colorectal Cancer Screening Among 986 Patients

Factor	Multivariable OR (95% CI)	*P* Value
Age, deciles	1.05 (0.90-1.24)	.53
Female	0.97 (0.59-1.58)	.89
Race/ethnicity		
White	1 [Reference]	NA
Black	0.99 (0.66-1.48)	.96
Latino	0.93 (0.45-1.89)	.98
Other	0.47 (0.20-1.10)	.08
Health literacy: comfort with forms^19^	1.01 (0.89-1.13)	.93
Trust in physician	1.19 (1.07-1.32)	.001
Perceived		
Health	1.41 (1.23-1.61)	<.001
Effectiveness of screening	0.86 (0.80-0.94)	<.001
Barriers to screening	1.20 (1.11-1.30)	<.001
Threat of CRC	0.81 (0.73-0.89)	<.001

Abbreviations: CRC, colorectal cancer; NA, not applicable; OR, odds ratio.

## Discussion

Screening is effective in lowering the incidence and mortality of CRC. However, prior data suggest that many older adults with comorbidities, limited life expectancy, and previous normal screening results may not benefit from ongoing screening colonoscopies.^1,5,6,7,8,9,14,15^ Moreover, these patients may be at increased risk for screening-related harms and burdens. Thus, experts recommend that screening decisions be individualized in older adults.

Despite these recommendations, our data suggest that many veterans would not be comfortable with cessation of CRC screening in the context of low value or with the use of risk calculators to guide this decision. These results are concerning as we move toward more personalized precision health approaches to prevention that incorporate individual patient characteristics to predict benefit and harm. However, our findings also indicate that patients have diverse opinions on this topic, because a substantial proportion of patients reported that they would be comfortable with screening cessation. In multivariable analysis, health belief model constructs, self-reported health, and trust in physician were associated with greater comfort, whereas demographic factors and health literacy were not. These data suggest that patients’ underlying mental models about screening and prognostication are determinants of their decision making in this context. Addressing misconceptions in these mental models through systematic and carefully designed educational interventions (such as decision aids) could improve patients’ understanding of the benefits and harms of low-value screening.^22^ Encouraging trusted clinicians to engage their patients in discussions around screening cessation could also be worthwhile. However, it is important to recognize that many patients conceptualize screening in a fundamentally different way than experts conceptualize screening. This difference is in part attributable to long-standing efforts to convince the public of the benefits of screening.^23^ However, the benefit of screening for a given patient is not static; it varies over the course of the patient’s life span, a nuance that has not been articulated to many patients and that runs counter to the widespread belief that prevention is always good. The concept of how screening benefit declines with advancing age and varies widely from patient to patient according to other factors should ideally be introduced earlier in the screening life cycle, when patients’ mental models about cancer prevention are more malleable. Addressing deep-seated misconceptions about screening through brief educational interventions and patient-clinician interactions later in life may prove to be challenging.

Our findings are consistent with the existing literature on attitudes toward cancer screening cessation. For example, a national survey in the United Kingdom that aimed to assess public attitudes toward cancer screening found that 90% of people believed that screening is almost always a good idea, and 49% stated that they would be tested for cancer even if it were untreatable.^24^ A survey in the United States that assessed public attitudes found similar results.^23^ In another study, Lewis et al^25^ surveyed 116 residents of 2 long-term care retirement communities. The authors found that 62% of participants believed that life expectancy was not important when deciding whether to continue screening. The proportion was even higher among those 85 years or older. Furthermore, 43% of participants stated that they would continue screening even if their physician recommended against it. Unlike these prior surveys, our study assessed attitudes toward screening cessation and toward the use of quantitative risk calculators. Moreover, the large sample size allowed us to explore associations between a variety of baseline characteristics (such as demographic factors and perceptions about screening) and attitudes toward screening cessation.

The reasons that patients wish to continue screening, even when the benefit may be low, are complex and may represent more of an emotional decision than a logical one. Such influences may include moral obligation (ie, the belief that it would be irresponsible to stop), knowledge seeking (ie, to gather information to plan ahead or for reassurance), established habits toward preventive care, prognostic skepticism (ie, the belief that statistics do not apply to the individual and the inability of physicians and experts to predict the future), and overall lack of interest or discomfort in discussing life expectancy.^22,23,24,25,26,27,28^ Our study is unique in that it assessed comfort with individualization using life expectancy calculators and CRC risk calculators specifically. As such tools become increasingly available and precision health interventions become more prominent, understanding and addressing barriers to uptake of such recommendations will become more important.^29^

### Strengths and Limitations

Strengths of our study include the high response rate (74%), which minimizes the potential for nonresponse bias; the large sample size (1415 respondents); and our survey design approach (which included semistructured interviews to inform survey development and piloting to refine the survey instrument before broader distribution). Of note, nearly one-third of the sample had limited or marginal health literacy, which differs from most prior studies focusing on more educated and affluent populations.

Our study also had several limitations. We examined the attitudes and preferences of veterans from a single region. These patients may differ from the general US population, especially because the majority of the respondents were white men. It is possible that women and minorities in the United States may respond differently to this survey. In addition, the respondents of our survey were individuals who had previously consented to colonoscopy and may have stronger preferences to continue colonoscopic CRC screening compared with those who have chosen other CRC screening modalities (such as stool tests) or have never been screened. Moreover, unmeasured factors, such as religious beliefs and cognitive function, could have influenced respondents’ attitudes toward continued low-value CRC screening. We were also limited in assessing the association between age and our primary outcome because we collected age in deciles rather than as a continuous variable (birth year). In addition, our study used a hypothetical survey that assessed patients’ attitudes and perceptions and is subject to social desirability. It is possible that actual behavior and action will differ in real-world settings.

## Conclusions

The findings of our study suggest that many veterans are resistant to low-value CRC screening cessation and are skeptical about a physician’s ability to predict CRC risk and life expectancy even with the use of risk calculators. Thus, efforts to tailor screening recommendations may be met with resistance from many patients. Future research is needed to better understand how to effectively communicate with patients on this topic.

## Notes

Supplement. **eAppendix.** Final Survey Instrument **eTable 1.** Attitudes Toward Using Age to Decide When to Start/Stop Colorectal Cancer Screening **eTable 2.** Attitudes Toward Using Risk Calculators to Inform Colorectal Cancer Screening Decisions **eTable 3.** Attitudes Toward Stopping Low-Value Colorectal Cancer Screening and Likelihood to Follow Recommendations to Stop Colorectal Cancer Screening
