# Supplementary material for: Patient Attitudes Toward Individualized Recommendations to Stop Low-Value Colorectal Cancer Screening
Source: JAMA Netw Open. 2018 Dec 7;1(8):e185461. doi: 10.1001/jamanetworkopen.2018.5461 (PMC6324357; doi:10.1001/jamanetworkopen.2018.5461)
Supplement: Supplement. — eAppendix. Final Survey Instrument eTable 1. Attitudes Toward Using Age to Decide When to Start/Stop Colorectal Cancer Screening eTable 2. Attitudes Toward Using Risk Calculators to Inform Colorectal Cancer Screening Decisions eTable 3. Attitudes Toward Stopping Low-Value Colorectal Cancer Screening and Likelihood to Follow Recommendations to Stop Colorectal Cancer Screening [file jamanetwopen-1-e185461-s001.pdf]

Supplementary Online Content

Piper MS, Maratt JK, Zikmund-Fisher BJ, et al. Patient attitudes toward individualized recommendations to stop low-value colorectal cancer screening. *JAMA Netw Open.* 2018;1(8):e185461. doi:10.1001/jamanetworkopen.2018.5461

eAppendix. Final Survey Instrument

eTable 1. Attitudes Toward Using Age to Decide When to Start/Stop Colorectal Cancer Screening

eTable 2. Attitudes Toward Using Risk Calculators to Inform Colorectal Cancer Screening Decisions

eTable 3. Attitudes Toward Stopping Low-Value Colorectal Cancer Screening and Likelihood to Follow Recommendations to Stop Colorectal Cancer Screening

This supplementary material has been provided by the authors to give readers additional information about their work.

© 2018 Piper MS et al. *JAMA Network Open.*

eAppendix. Final Survey Instrument

Dear Veteran,

This survey is part of a research project of the Health Services Research and Development (HSR&D) Center for Clinical Management Research at the Ann Arbor VA Medical Center. **This survey asks about your beliefs and opinions about personalized screening for colon cancer, including how you feel about stopping screening in some instances. You will learn about a hypothetical (not real) calculator that predicts your chances of getting colon cancer.**

It will take about 15-20 minutes to complete the survey. As you go through the survey, please do not read ahead or go back and change your answers. Your answers will help us understand the best way to talk to patients like you who might face these situations in real life in the future.

Even though we ask you questions about hypothetical situations, some of the information in this survey may relate to your life. If you have questions or concerns about your personal health, please talk to your doctor.

All of your responses will be kept completely anonymous and confidential. Your participation is completely voluntary, and you will not lose any of your benefits if you choose not to take our survey. If you do want to take our survey, you can stop at any time or choose not to answer any of the questions.

If you have questions or concerns about this survey please contact:

Sameer D. Saini, MD, MS (Principal Investigator)
Health Services Research & Development (HSR&D)
VA Center for Clinical Management Research
VA Ann Arbor Healthcare System
(734) 845-5865

Thank you for taking the time to complete this survey.

Sincerely,

Sameer D. Saini, MD, MS

© 2018 Piper MS et al. *JAMA Network Open.*

To start off, we'd like to tell you a little about colon polyps, colon cancers, and colonoscopies.

Colon cancer is a medical condition where a tumor or growth forms in the lining of the colon, part of the digestive system where stool or poop is made. Colon cancer is the second-leading cause of cancer deaths in the United States, making it a common cancer. If we find colon cancer at an early stage *before* symptoms start, it's usually curable. If we detect it at a later stage, *after* symptoms have already started (like blood in the stool or belly pain), it's a lot harder to treat.

Most colon cancers start out as small growths in the lining of the colon, called **colon polyps.** Patients who have colon polyps are more likely to get colon cancer than those who don't. However, even patients who have colon polyps don't usually get colon cancer.

Screening is when we look for cancer or polyps in someone who doesn't have any symptoms. Experts agree that screening for colon cancer can be beneficial because it detects cancer early when it can be cured. However, most people will never get colon cancer. This means that most patients who are screened don't get any personal benefit from screening even though they go through all of the effort.

A **colonoscopy** is a type of screening test in which doctors insert a flexible tube into the colon to look for cancer or polyps. To get a colonoscopy, you first need to drink a "prep" solution to clean out your colon the day before the procedure. This "prep" gives you severe diarrhea and is usually the part patients dislike the most. On the day of the procedure, you get an intravenous line ("IV") and are given some medication to make you sleepy and comfortable for the procedure. Most patients don't remember much about the actual procedure.

If the doctor gets a good, clear look at the lining of your whole colon and doesn't see any polyps or growths, your colonoscopy is considered "normal". This is the case with most patients. Right now, doctors will usually recommend that someone with a normal colonoscopy get a repeat colonoscopy in 10 years. Unfortunately, we don't know if this is the best thing for all patients.

© 2018 Piper MS et al. *JAMA Network Open.*

AS YOU GO THROUGH THE REST OF THE SURVEY, PLEASE DO NOT READ AHEAD OR GO BACK AND CHANGE YOUR ANSWERS.

© 2018 Piper MS et al. *JAMA Network Open.*

Now imagine how you'd feel in the following situation…

Let's say that you're feeling fine, but you go to see your regular doctor for a routine check-up. **You have been seeing this same doctor for many years, and you trust his medical opinion.** Your doctor first talks to you about what's going on with your health. He then starts to talk to you about ways to STAY as healthy as possible. **The doctor tells you that he is aware that you had a screening colonoscopy 10 years ago.** According to his records, **the colonoscopy did not show any polyps.**

The doctor reminds you that **these days, experts recommend that patients with no family history of colon cancer get their first screening colonoscopy at age 50**. He goes on to explain that the *reason* they recommend starting at age 50 (and not at a younger age) is because colon cancer usually does not occur in younger people without a family history of colon cancer.

Given what you've just learned:

1. How **reasonable** does it seem to you that experts recommend using age to decide when to start screening?

   ☐0 ☐1 ☐2 ☐3 ☐4 ☐5 ☐6

   Not At All Reasonable … Extremely Reasonable

2. How **reasonable** does it seem to you that experts recommend starting screening **at the same age for all patients** who have no family history of colon cancer?

   ☐0 ☐1 ☐2 ☐3 ☐4 ☐5 ☐6

   Not At All Reasonable … Extremely Reasonable

3. At **what age** do YOU think doctors should start screening for colon cancer in patients who have no family history?

   ☐ Before age 50
   ☐ At age 50
   ☐ After age 50
   ☐ Doctors should **not** use age to decide when to start screening (they should use other factors instead).

© 2018 Piper MS et al. *JAMA Network Open.*

The doctor goes on to tell you that **experts are also recommending that patients who had a normal colonoscopy in the past should STOP getting routine screening for colon cancer at age 75.** This is because it takes about 10 years for a colon polyp to become a colon cancer. So, people who are 75 years old and have never had colon polyps before often don't live long enough to develop a colon cancer.

<u>Given what you've just learned:</u>

4. How **reasonable** does it seem to you that experts recommend using age to decide when to STOP screening?

☐0 ☐1 ☐2 ☐3 ☐4 ☐5 ☐6

Not At All Reasonable — Extremely Reasonable

5. How **reasonable** does it seem to you that experts recommend stopping routine screening **at the same age for all patients who have previously had a normal screening colonoscopy?**

☐0 ☐1 ☐2 ☐3 ☐4 ☐5 ☐6

Not At All Reasonable — Extremely Reasonable

6. At **what age** do YOU think doctors should stop screening patients who have had a normal screening colonoscopy in the past?
   - ☐ Before age 75
   - ☐ At age 75
   - ☐ After age 75
   - ☐ Doctors should **not** use age to decide when to stop screening (they should use other factors instead).

7. If your health continues to be pretty much the same as it is now, at **what age** would you be **comfortable** with NOT getting any more screening colonoscopies (if you had already had a normal screening colonoscopy in the past)?
   - ☐ Before age 75
   - ☐ At age 75
   - ☐ After age 75
   - ☐ I would **never** be comfortable stopping due to my age.

© 2018 Piper MS et al. *JAMA Network Open.*

Now imagine that the doctor tells you that even though most 75 year-olds who have no colon polyps won't live long enough to develop colon cancer, **some people will live longer than average.** In other words, some 75 year-olds will live to be 100 years old. These patients might benefit from screening after age 75.

On the other hand, some 65 year-olds have serious health problems that shorten their life span. Because they're sick, these patients might not live more than a few years. And because colon cancer takes many years to develop, these patients are unlikely to develop cancer in their lifetimes. So, screening is unlikely to benefit them.

In other words, the doctor says, **every patient is different.** If we stop screening **very healthy** people at age 75, this might not be the right thing for them since they could live many more years. On the other hand, if we keep screening **unhealthy** people until age 75, this might not be the right thing for them either. **What we need to do is to screen patients only if they are likely to live long enough to benefit.**

Now imagine that doctors have a special kind of **life expectancy calculator** that can estimate how long you will live. This calculator, which is based on scientific research studies, weighs information like your age and your overall health status. **Doctors can use this life expectancy calculator to help them decide whether YOU should keep getting screened for colon cancer (or you can stop).**

<u>Given what the doctor has told you:</u>

8. How **reasonable** does it seem to you that doctors would use a calculator that predicts life expectancy to decide whether to stop screening?

   ☐0 ☐1 ☐2 ☐3 ☐4 ☐5 ☐6

   Not At All Reasonable — Extremely Reasonable

9. How **accurate** do you think a life expectancy calculator like this would be?

   ☐0 ☐1 ☐2 ☐3 ☐4 ☐5 ☐6

   Not At All Accurate — Extremely Accurate

10. If you personally had **serious health problems** that were likely to shorten your life, and your doctor did not think screening would be of much benefit based on the calculator, how **comfortable** would you be with not getting any more screening colonoscopies?

    ☐0 ☐1 ☐2 ☐3 ☐4 ☐5 ☐6

    Not At All Comfortable — Extremely Comfortable

© 2018 Piper MS et al. *JAMA Network Open.*

Now imagine that the doctor tells you that **even if you are healthy and will live for many years, screening STILL might not benefit you.** This is because some patients are not very likely to get colon cancer. We call these patients “low risk”. In the past, we didn’t know which patients were low risk for colon cancer, so we recommended that EVERYONE come back for repeat screening every 10 years. **Based on scientific research, we can now figure out if YOU are a low risk patient who might not need any more screening.**

To predict your risk for colon cancer, the doctor tells you that he will use your age, your family history, what you eat, your weight, whether or not you smoke, and the results of your last colonoscopy. He’ll put this information into a special **colon cancer risk calculator** that figures out your personal chances of getting colon cancer. This **colon cancer risk calculator** considers much more information than the life expectancy calculator you learned about earlier.

The doctor tells you that he’ll use the calculator results to recommend whether YOU should get another screening colonoscopy. **If you are low risk according to the colon cancer risk calculator, you might not need any more screening.** The doctor tells you that the approach he’s using is recommended by experts.

<u>Given what the doctor has told you:</u>

11. How **reasonable** does it seem to you that doctors would use a calculator that predicts colon cancer risk to decide whether to stop screening?

☐0 ☐1 ☐2 ☐3 ☐4 ☐5 ☐6

Not At All Reasonable ... Extremely Reasonable

12. How **accurate** do you think a colon cancer risk calculator like this would be?

☐0 ☐1 ☐2 ☐3 ☐4 ☐5 ☐6

Not At All Accurate ... Extremely Accurate

13. How **comfortable** are you with your doctor using the colon cancer risk calculator he just described to decide whether or not you should get another screening colonoscopy?

☐0 ☐1 ☐2 ☐3 ☐4 ☐5 ☐6

Not At All Comfortable ... Extremely Comfortable

© 2018 Piper MS et al. *JAMA Network Open.*

Now imagine that, as you sit in his office, the doctor uses a colon cancer risk calculator to figure out your chances of getting colon cancer. He turns to you and tells you that your risk of getting colon cancer is low – **low enough that he does not think another screening colonoscopy is necessary**.

The doctor tells you that this risk calculation can be done again at future visits, though he doesn't think the results will change.

The doctor finishes up by reminding you that this does NOT mean that you have NO chance of getting colon cancer. In fact, he says, you could still get cancer, but it's very unlikely. So a screening colonoscopy is probably not worth the hassle, and he does NOT recommend it.

Given everything you've learned so far:

14. If you were the patient in this scenario, how **comfortable** would you be with the doctor's recommendation to stop screening for colon cancer?

☐0 ☐1 ☐2 ☐3 ☐4 ☐5 ☐6

Not At All Comfortable — Extremely Comfortable

15. In the end, how **likely** do you think you'd be to follow the doctor's recommendation to stop screening for colon cancer (whether or not you are comfortable with it)?

☐0 ☐1 ☐2 ☐3 ☐4 ☐5 ☐6

Not At All Likely — Extremely Likely

© 2018 Piper MS et al. *JAMA Network Open.*

Colonoscopy appointments are in short supply. Even though we'd like to, we can't do a screening colonoscopy on every veteran right away because there just aren't enough doctors to do all of the procedures. This means that patients sometimes have to wait up to a few months for their colonoscopy. Patients who have higher risk for colon cancer have to wait just like everyone else.

Given what you've just learned about colonoscopies being in short supply:

16. How **reasonable** does it seem to you that patients would have to wait to get their screening colonoscopy on a first come, first serve basis?

☐0 ☐1 ☐2 ☐3 ☐4 ☐5 ☐6

Not At All Reasonable — Extremely Reasonable

17. If you were at **lower risk** for cancer than other patients, would you be **willing** to wait up to 6 months to get your next screening colonoscopy, so that higher risk patients could be screened first?
    - ☐ Definitely Would Be Willing to Wait
    - ☐ Probably Would Be Wiling to Wait
    - ☐ Probably Would NOT Be Willing to Wait
    - ☐ Definitely Would NOT Be Willing to Wait

© 2018 Piper MS et al. *JAMA Network Open.*

Questions About You:

Now we will ask you a few questions about you.

18. What is your age?
    - ☐ 18-29
    - ☐ 30-39
    - ☐ 40-49
    - ☐ 50-59
    - ☐ 60-69
    - ☐ 70-79
    - ☐ 80-89
    - ☐ 90 or above

19. What is your gender? ☐ Male ☐ Female ☐ Other/Transgender

20. What is the **highest** grade or level of schooling and/or training you completed? *(check one*)
    - ☐ Less than 8 years
    - ☐ 8-11 years
    - ☐ 12 years or completed high school or GED
    - ☐ Vocational, technical, or business training
    - ☐ 1-3 years of college/junior/community college
    - ☐ 4 or more years of college or graduated from college
    - ☐ Graduate or professional school
    - ☐ Other (please specify): ________________________________

21. What is your current marital status? *(check one*)
    - ☐ Married, or living as married
    - ☐ Widowed
    - ☐ Divorced
    - ☐ Separated
    - ☐ Never married

© 2018 Piper MS et al. *JAMA Network Open.*

Questions About You:

22. Do you consider yourself to be Hispanic or Latino? (*check one*)
    ☐ Yes ☐ No

23. Do you consider yourself to be of Middle Eastern or Arab origin? (*check one*)
    ☐ Yes ☐ No

24. Which of these best describes your race? NOTE: Mark all that apply.
    ☐ White
    ☐ Black or African American
    ☐ Asian
    ☐ Native Hawaiian or other Pacific Islander
    ☐ American Indian or Alaskan Native
    ☐ Other (please specify): ____________________________

25. In general, would you say your health is *(check one)*:
    ☐ Excellent
    ☐ Very good
    ☐ Good
    ☐ Fair
    ☐ Poor

For the next set of questions we will ask you about your experience with colon cancer and screening. **If you are not sure of an answer please give your best guess.**

**26-28. When was the last time you had a …**	Within the last year	Between 1 and 5 years ago	Between 5 and 10 years ago	More than 10 years ago	Never
Colonoscopy?	☐	☐	☐	☐	☐
Sigmoidoscopy?	☐	☐	☐	☐	☐
Test for blood in the stool ("stool cards," "stool vials," or FOBT)?	☐	☐	☐	☐	☐

© 2018 Piper MS et al. *JAMA Network Open.*

**29-32. Have you ever had or been told you had…**	Yes	No
Blood in your stool?	☐	☐
Colon polyps?	☐	☐
Colon cancer?	☐	☐
A family history of colon cancer? (one or more close relatives diagnosed with colon cancer)	☐	☐

33. If you have never had colon cancer, how **likely** do you think you are to get colon cancer sometime in your lifetime?

☐0 ☐1 ☐2 ☐3 ☐4 ☐5 ☐6

Not At All Likely … Extremely Likely

34. If you have never had colon cancer, how **worried** are you about getting colon cancer sometime in your lifetime?

☐0 ☐1 ☐2 ☐3 ☐4 ☐5 ☐6

Not At All Worried … Extremely Worried

35. How **effective** do you think getting regular colonoscopies is in reducing your risk of colon cancer?

☐0 ☐1 ☐2 ☐3 ☐4 ☐5 ☐6

Not At All Effective … Extremely Effective

36. How **hard** is it for you to schedule and travel to a colonoscopy appointment?

☐0 ☐1 ☐2 ☐3 ☐4 ☐5 ☐6

Not At All Hard … Extremely Hard

37. How **hard** is it for you to deal with getting a colonoscopy itself (including the bowel “prep”)?

☐0 ☐1 ☐2 ☐3 ☐4 ☐5 ☐6

Not At All Hard … Extremely Hard

© 2018 Piper MS et al. *JAMA Network Open.*

38. Overall, how would you rate your **knowledge** of colon cancer before taking this survey?

☐0 ☐1 ☐2 ☐3 ☐4 ☐5 ☐6

Did Not Know Anything About Colon Cancer — Knew A Lot About Colon Cancer

For the next set of questions we will ask you about **your experience with health-related information.**

39. How often do you have someone (like a family member, friend, hospital/clinic worker, or caregiver) help you read hospital materials?
    - ☐ Always
    - ☐ Often
    - ☐ Sometimes
    - ☐ Occasionally
    - ☐ Never

40. How confident are you filling out medical forms by yourself?
    - ☐ Extremely
    - ☐ Quite a bit
    - ☐ Somewhat
    - ☐ A little bit
    - ☐ Not at all

41. How often do you have problems learning about your medical condition because of difficulty understanding written information?
    - ☐ Always
    - ☐ Often
    - ☐ Sometimes
    - ☐ Occasionally
    - ☐ Never

42. In general, how **likely** are you to trust your doctor's medical recommendations?

☐0 ☐1 ☐2 ☐3 ☐4 ☐5 ☐6

Not At All Likely — Extremely Likely

© 2018 Piper MS et al. *JAMA Network Open.*

Thank you for participating in this survey!

© 2018 Piper MS et al. *JAMA Network Open.*

eTable 1. Attitudes Toward Using Age to Decide When to Start/Stop Colorectal Cancer Screening

**Questions (see supplemental for full questions)**	**Total Participants in survey, No. (%)**
"How reasonable does it seem to you that experts recommend using age to decide when to start screening?"	
1 (Not at all)	50 (4.9)
2	20 (1.9)
3	38 (3.7)
4	125 (12.1)
5	166 (16.1)
6	285 (27.6)
7 (Extremely)	347 (33.7)
"How reasonable does it seem to you that experts recommend using age to decide when to STOP screening?"	
1 (Not at all)	227 (22.0)
2	76 (7.4)
3	74 (7.2)
4	159 (15.4)
5	128 (12.4)
6	198 (19.2)
7 (Extremely)	171 (16.6)
"At what age do YOU think doctors should stop screening patients who have had a normal screening colonoscopy in the past?"	
Before 75	65 (6.3)
At 75	278 (26.9)
After 75	180 (17.4)
Should never use age	509 (49.3)

© 2018 Piper MS et al. *JAMA Network Open.*

eTable 2. Attitudes Toward Using Risk Calculators to Inform Colorectal Cancer Screening Decisions

**Questions (see supplemental for full questions)**	**Total Participants in survey, No. (%)**
"How reasonable does it seem to you that doctors would use a calculator that predicts life expectancy to decide whether to stop screening?"	
1 (Not at all)	332 (31.7)
2	87 (8.3)
3	105 (10.0)
4	144 (13.7)
5	136 (13.0)
6	130 (12.4)
7 (Extremely)	115 (11.0)
"How reasonable does it seem to you that doctors would use a calculator that predicts colon cancer risk to decide whether to stop screening?"	
1 (Not at all)	255 (24.3)
2	97 (9.3)
3	101 (9.6)
4	159 (15.2)
5	164 (15.6)
6	154 (14.7)
7 (Extremely)	119 (11.3)

© 2018 Piper MS et al. *JAMA Network Open.*

eTable 3. Attitudes Toward Stopping Low-Value Colorectal Cancer Screening and Likelihood to Follow Recommendations to Stop Colorectal Cancer Screening

**Questions (see supplemental for full questions)**	**Total Participants in survey, No. (%)**
"If you personally had serious health problems that were likely to shorten your life, and your doctor did not think screening would be of much benefit based on the calculator, how comfortable would you be with not getting any more screening colonoscopies?" (Primary Outcome)	
1 (Not at all)	300 (28.7)
2	88 (8.4)
3	89 (8.5)
4	146 (13.9)
5	121 (11.6)
6	166 (15.9)
7 (Extremely)	137 (13.1)
"In the end, how likely do you think you'd be to follow the doctor's recommendation to stop screening for colon cancer (whether or not you are comfortable with it)?"	
1 (Not at all)	298 (28.8)
2	98 (9.5)
3	99 (9.6)
4	138 (13.3)
5	131 (12.6)
6	138 (13.3)
7 (Extremely)	134 (12.9)

© 2018 Piper MS et al. *JAMA Network Open.*
